# Pure laparoscopic hepatectomy in semiprone position for right hepatic major resection

**DOI:** 10.1007/s00534-012-0558-y

**Published:** 2012-10-02

**Authors:** Tetsuo Ikeda, Yohei Mano, Kazutoyo Morita, Naotaka Hashimoto, Hirohito Kayashima, Atsuro Masuda, Toru Ikegami, Tomoharu Yoshizumi, Ken Shirabe, Yoshihiko Maehara

**Affiliations:** Department of Surgery and Science, Graduate School of Medical Sciences, Kyushu University, 3-1-1 Maidashi, Higashi-ku, Fukuoka, 812-8582 Japan

**Keywords:** Pure laparoscopic hepatectomy, Semiprone position, Anatomical liver resection, Rouviere’s sulcus

## Abstract

**Background:**

Pure laparoscopic liver resection is technically difficult for tumors located in the dorsal anterior and posterior sectors. We have developed a maneuver to perform pure laparoscopic hepatectomy in the semiprone position which was developed for resecting tumors located in these areas.

**Methods:**

The medical records have been reviewed retrospectively in 30 patients who underwent laparoscopic liver resection in the semiprone position for carcinoma in the dorsal anterior or posterior sectors of the right liver between 2008 and 2011.

**Results:**

Seventeen liver tumors were primary liver tumors and 13 were colorectal metastases. Of the 30 patients, 11 (36.6 %) underwent major hepatectomy [right hemihepatectomy in 7 (23.3 %) and posterior sectionectomy in 4 (13.3 %)]. Anatomical minor resection, such as S6 or S7 segmentectomy, was performed in five patients (16.6 %). Five patients with liver metastasis underwent a simultaneous laparoscopic resection. There was no mortality, reoperation, or conversion to open procedures. There were no hepatectomy-related complications such as postoperative bleeding, bile leakage, or liver failure.

**Conclusions:**

Pure laparoscopic hepatectomy in the semiprone position for tumors present in the dorsal anterior and posterior sectors is feasible and safe. This method expands the indications for laparoscopic liver resection for tumors.

**Electronic supplementary material:**

The online version of this article (doi:10.1007/s00534-012-0558-y) contains supplementary material, which is available to authorized users.

## Introduction

In November 1994, laparoscopic hepatectomy was introduced to our institution on the principle that parenchymal division would be performed under direct vision through a small laparotomy wound. In 1996, pure laparoscopic partial hepatectomy with parenchymal division using a linear stapler was also introduced.

In June 2008, pure laparoscopic hepatectomy (PLH) involving hepatic parenchymal division of most of the liver was performed with a TissueLink Monopolar Sealer (TL-MS; Endo SH2.0™ sealing hook) and a laparoscopic Cavitron ultrasonic dissector (CUSA; Radionics, Burlington, MA, USA). First, the liver parenchyma was coagulated with the TL-MS, and the coagulated liver parenchyma was emulsified and fractured with the CUSA. We managed to perform pure laparoscopic partial hepatectomy for tumors located at the inferior edge of the liver and left lateral segmentectomy in a safe, stable manner. Even when the tumor was present on the liver surface, PLH of the dorsal part of the anterior sector and posterior sector was necessary to mobilize the liver from the inferior vena cava (IVC). When tumors were located on the lateral side of the right liver, the patient was placed in the left lateral position, and the position of the tumor moved to the top of the field. Furthermore, for tumors located on the dorsal aspect of the right liver, better results were obtained when the patient was tilted more toward the prone position. Next, pure laparoscopic right lobectomy, which requires complete mobilization of the right liver lobe from the IVC and hepatic vascular exclusions, was attempted [1].

Manipulation in the semiprone position has proved to be very useful during mobilization of the right liver for pure laparoscopic right hepatectomy. Mobilization of the right liver requires dissection of the short hepatic vein that directly branches from the IVC, divides the IVC ligament, and further encircles the right hepatic vein. When the patient is in either a supine or semilateral position, complete mobilization of the right liver requires lifting the heavy, fragile right hepatic lobe. If the liver is excessively elevated to expand the view, there is a risk of injuring the short hepatic veins or IVC itself. Furthermore, once bleeding occurs around the IVC, it is very difficult to identify the bleeding source and place sutures to stop the bleeding while lifting the bent-over right lobe and suctioning the blood. In contrast, in the semiprone position, even if bleeding has occurred, it is not necessary to lift the heavy right lobe because the blood will generally flow to the lower left side; therefore, reliable hemostasis can be achieved after identifying the bleeding source using both hands of the operator. Initially, exclusion of portal pedicles at the hepatic hilum is difficult if the patient is kept in the semiprone position. In fact, the organs surrounding the hepatoduodenal ligament, such as the stomach, duodenum, and colon, also hang down toward the left leg together with the liver when the patient is in the semiprone position. This allows for a sweeping view of the main portal fissure, right portal fissure, and portal fissure of the caudate process, which is necessary when performing the Glissonian approach to the right lobe.

Therefore, PLH in the semiprone position is indicated under the following conditions: partial resection of the tumor located in S6, S7, and the dorsal area of S8; anatomical segmentectomy of S6 and S7; posterior sectionectomy; and right hemihepatectomy.

## Methods

### Patients

A total of 125 laparoscopic hepatectomy procedures were performed between November 1994 and November 2011 in two institutions of our center. In 46 of the 125 patients, the tumor was located in the dorsal part of the anterior sector and posterior sector. Laparoscopic hepatectomy in the semiprone position has been used since February 2010. A total of 30 laparoscopic hepatectomy procedures were performed in the semiprone position.

The indications for laparoscopic liver resection were similar to those for open liver resection with respect to preoperative assessment of liver function, type of resection, and postoperative care. The standard preoperative investigations included liver imaging [spiral computed tomography (CT) and contrast ultrasonography as routine procedures, magnetic resonance imaging and positron emission tomography if required, chest imaging (plain X-ray or CT), and clinical biochemistry]. To determine the method (laparoscopy vs. open) and extent of resection, all patients with hepatocellular carcinoma (HCC) underwent preoperative examinations that included assessments of liver function reserve (liver function tests, Child–Pugh classification, and indocyanine green retention rate at 15 min).

### Surgical technique

#### PLH in the semiprone position

Pneumoperitoneum was established at a pressure of 8 mmHg, and four trocars were inserted below the costal arch from the right linea axillaris media to the midline. Intraoperative hemodynamic monitoring of systemic arterial pressure and central venous pressure was routinely performed for all patients.

**Fig. 1:**
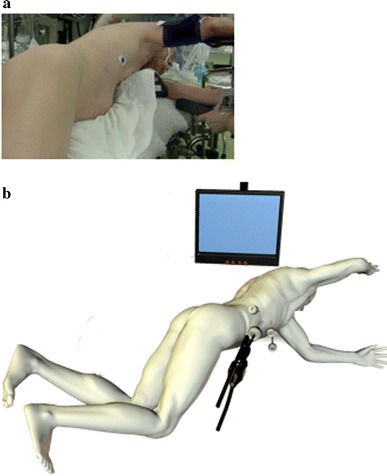
**a** Patient position immediately before surgery. The semiprone position is similar to the position during breathing while swimming. **b** Illustration of patient position and trocar placement. Four trocars (three 12-mm and one 5-mm) are placed in the right lateral region and below the costal arch from the right linea axillaris media to the midline

The patient was placed in a semiprone position, which is similar to the position during breathing while swimming the crawl (Fig. 1a). The surgeon was positioned on the patient’s left cranial side, and the camera operator was positioned on the patient’s left side. A laparoscopic trocar was inserted into the right lateral region, and then four trocars were inserted below the costal arch from the right middle axillary line to the pararectal line (Fig. 1b). Pneumoperitoneum was established by an open technique, and the intra-abdominal carbon dioxide gas pressure was set at 8–10 mmHg. A 30° laparoscope and 5- and 12-mm trocars were used. In this semiprone position, the liver naturally slips to the left lower quadrant, and the large working space can be used for hepatectomy in the right subphrenic area. With the patient in this position, complete resection consisted of the following three steps. First, Rouviere’s sulcus (a cleft in the liver running to the right of the hilum and a landmark demarcating the division between S6 and S5) was identified, and the posteroinferior Glisson’s sheath, which runs behind Rouviere’s sulcus, was isolated. After division of a substantial amount of hepatic tissue along Glisson’s sheath, the hepatic pedicle structures (anterior and posterior pedicles and S6, S7, and S8 pedicles and their branches) were isolated within the liver parenchyma. For selective inflow occlusion, the intrahepatic portion of the Glissonian pedicle toward the lesion or area to be removed was encircled with tape and clamped. To verify whether the tumors were included in the ischemic areas, the liver was thoroughly examined using laparoscopic Doppler ultrasonography. After the ischemic demarcation line had been clearly observed along the planned resection line, the clamped Glissonian pedicles were usually divided with Hem-o-lok clips.

Second, sufficient amounts of the right triangular and coronary ligaments were divided. The IVC, right inferior hepatic vein, and short hepatic vein were also carefully divided. During liver mobilization, the right liver lobe position could be controlled with only a 5-mm pledget operated by an assistant.

Finally, parenchymal division was performed using an EnSeal^®^ (Ethicon Endo-Surgery, Cincinnati, OH, USA) and water-dripping bipolar forceps. The liver surface was quickly and easily divided with the EnSeal, which coagulates attached tissue with a single straight jaw. At the start of the coagulation, the straight jaw was inserted into the shallowest portion of the liver, and the jaws were closed. The tissue sandwiched between both jaws was sealed and divided. This manipulation was repeated in the same plane until the desired site was reached. It was then repeated for the next layers working deeper. However, the central liver parenchyma, especially near the hepatic vein, bleeds easily. In such areas, forceps with a water-dripping function are useful. This instrument washes the blood away and coagulates the tissue. Bleeding may unexpectedly occur during dissection around the IVC. In these cases, small perforating vessels or accessory hepatic veins can be perforated. If the patient is placed in the semiprone position, a hemostatic suture can be easily placed with both hands because of the good field of view without strong liver traction. After completion of the parenchymal division, the resected specimen was placed in a plastic bag. The specimen was extracted through the trocar site in the right lateral region, which was expanded to the proper size.

We performed laparoscopic posterior segmentectomy in the semiprone position (see Video, Supplemental Digital Content 1).

The right lobectomy technique has been described in detail [1]. In right lobectomy, mobilization of right liver from the IVC must first be performed up to the bifurcation of the right hepatic vein. Using the Endo Mini-Retract™ (United Surgical, a division of Tyco Healthcare Group LP, Norwalk, CT, USA), the right hepatic vein was scooped up and encircled with vessel tape. Second, inflow occlusion was performed using an extrahepatic Glissonian approach. The parenchymal division was continued along the right border of the right hepatic vein. Finally, the origin of the right hepatic vein from the IVC was divided with a vascular stapler.

## Results

The indications for laparoscopic liver resection and the patient and tumor characteristics are summarized in Table 1. Seventeen liver tumors were primary liver tumors, 16 were HCC, and one was cholangiocarcinoma; 13 cases of colorectal metastases were also included. Most patients had underlying liver disease related to hepatitis B or C virus or preoperative chemotherapy. Twenty patients (66.6 %) had chronic hepatitis, and 12 (40.0 %) had liver cirrhosis; however, all patients were classified as Child–Pugh class A.

**Table 1:** Preoperative characteristics of patients

Factors	PLH-SP (*n* = 30)
Age [years; mean (range)]	66 (48–86)
Sex (M/F)	25/5
BMI (mean ± SE)	23.6 ± 2.9
Previous laparotomy [*n* (%)]	16 (57.1)
Preoperative chemotherapy [*n* (%)]	6 (21.4)
HBsAg (+) [*n* (%)]	3 (10.7)
Anti-HCV (+) [*n* (%)]	7 (25)
Liver disease (normal/CLD/LC)	6/15/9
Child–Pugh class (A/B/C)	30/0/0
ICG-R15 [*n* (%)]	15.6 ± 10.7
Indications for laparoscopic liver resection	
Primary liver tumors	17
Hepatocellular carcinoma	16
Cholangiocarcinoma	1
Metastatic tumors	13
Colorectal adenocarcinoma	13
Tumor characteristics	
Size [(cm); mean ± SE]	2.6 ± 1.0
Number (1/2/3)	20/8/2
Location (superficial/deep)	19/11

*PLH-SP* pure laparoscopic hepatectomy in semiprone position

Types of liver resections are listed in Table 2. Thirty-five liver resections were performed in 30 patients (3 double and 1 triple resection). Among the 30 patients, 11 (36.6 %) major hepatectomies [7 (23.3 %) right hemihepatectomies and 4 (13.3 %) posterior sectionectomies] were performed. Anatomical minor resection, such as S6 or S7 segmentectomy, was performed in five patients (16.6 %).

**Table 2:** Type of laparoscopic liver resection

Type	PLH-SP (*n* = 30)
Pure laparoscopic/laparoscopic assisted	27/3
Major liver resection (*n*)	11
Right hemihepatectomy (*n*)	7
Right posterior sectionectomy (*n*)	4
Minor liver resection (*n*)	26
Anatomical liver resection (*n*)	5
S6 segmentectomy (*n*)	3
S7 segmentectomy (*n*)	2
Non-anatomical liver resection (*n*)	21
S5 partial resection (*n*)	3
S6 partial resection (*n*)	3
S7 partial resection (*n*)	7
S8 partial resection (*n*)	8
Total	37

*PLH-SP* pure laparoscopic hepatectomy in semiprone position

Intraoperative and postoperative outcomes are summarized in Table 3. No conversions to open surgery occurred in either of the studied groups. Seven of 12 patients with metastatic colorectal cancer had undergone previous resection of a primary tumor. The remaining five patients with liver metastasis underwent a simultaneous laparoscopic resection such as low anterior resection (three patients) or right colectomy (one patient) for primary rectal and colon cancer. Another patient with HCC underwent laparoscopic distal gastrectomy because early gastric cancer was found during the preoperative examination for HCC.

**Table 3:** Surgical outcomes and histopathological data

Parameters	PLH-SP (*n* = 30)
Open conversion (*n*)	0
Simultaneous combined resection [*n* (%)]	5 (17.8)
Rectum [*n* (%)]	3 (10.7)
Right colon [*n* (%)]	1 (3.5)
Gastrectomy [*n* (%)]	1 (3.5)
Spleen (*n*)	0
Operative time [min; median (range)]	373 (79–881)
Without simultaneous G-I resection^a^ [min; median (range)]	301 (79–697)
Blood loss [ml; median (range)]	146 (0–550)
Without simultaneous G-I resection^a^	91 (0–330)
Blood transfusions [*n* (%)]	1 (3.3)
Postoperative complications [*n* (%)]	2 (6.6)
Without simultaneous G-I resection^a^ (*n*)	0
Intra-abdominal abscess [*n* (%)]	2 (6.6)
Ascites [*n* (%)]	0
Bile leakage [*n* (%)]	0
Postoperative hospital stay [days; median (range)]	16 (5–44)
Without simultaneous G-I resection^a^ [days; median (range)]	9 (5–15)
Histopathological data	
Tumor-free margin resection [*n* (%)]	30 (100)
Minimal distance from resection line to tumor tissue [mm; mean (range)]	5 (1–30)
Weight of resected specimen [g; median (range)]	269 (9–890)

Data are presented as median (range) or number (%)

*PLH-SP* pure laparoscopic hepatectomy in semiprone position

^a^Excluding the cases with simultaneous stomach or colorectal resection

The mean operation time was 373 min, which was considered to be long. However, when simultaneous colorectal and stomach resection was excluded, the mean operation time was 301 min. The mean blood loss was 146 g. However, when simultaneous colorectal and stomach resection was excluded, the mean blood loss was 91 g. Blood transfusion was required in only one case. The mean postoperative hospital stay was 16 days, but, again, when simultaneous colorectal and stomach resection was excluded, it was 9 days.

In this study, two patients (6.6 %) experienced postoperative complications. No cases of postoperative bleeding, bile leakage, or liver failure were observed. Intra-abdominal abscesses requiring treatment were observed in two patients who underwent simultaneous colorectal resection. In one of these cases, the abscess was found in the area of partial liver resection of S8 and was managed by percutaneous drainage and right colectomy. The other patient underwent rectal low anterior resection, and the abscesses were observed around the anastomoses and in the lower right abdomen.

There were no operative mortalities, reoperations, major complications, or episodes of gas embolism during or after the operations.

The histological results are presented in Table 3. Tumor-free margin resection was 100 %, the minimum distance from the resection line to tumor tissue was 5 mm, and the mean weight of the resected specimen was 269 g.

## Discussion

The liver lies mostly under the cover of the thoracic bony cage and is also covered by the diaphragm. Therefore, liver resection requires an extremely large abdominal incision and, in some cases, an additional chest wall incision.

PLH in the semiprone position is performed under the following conditions: partial resection of the tumor located in S6, S7, and the dorsal area of S8; anatomical segmentectomy of S6 and S7; posterior sectionectomy; and right hemihepatectomy. Some groups have reported the feasibility of pure laparoscopic liver resection for tumors located in the posterosuperior segments [2–4]. Kazaryan et al. [2] reported that although laparoscopic resection for posterosuperior segments has certain technical challenges, an appropriate adjustment of surgical techniques and optimal patient positioning enables this laparoscopic technique to provide safe and effective parenchyma-sparing resections for lesions located in both the posterosuperior and anterolateral segments.

HCC is the most common indication for laparoscopic hepatectomy. Major hepatectomy may be difficult because of basal chronic liver dysfunction. Hepatic colorectal cancer metastases are the next most common indication for laparoscopic hepatectomy. Recently, several multidisciplinary therapies have been used to increase the resectability rate for patients with initially nonresectable colorectal liver metastases. These therapies include transarterial embolization, ablative techniques, and potent systemic chemotherapy, which has been developed in recent years. On the other hand, these cases could indicate that major hepatectomy was relatively decreased because of patients with multiple tumors in both hepatic lobes and liver dysfunction secondary to preoperative therapies, especially potent systemic chemotherapy itself. Metastatic liver tumors are more likely to be found in the posterosuperior segments because of the liver volume.

We can make full use of the latest technology, devices, and innovations for PLH in resection of major hepatic tumors as follows:

The first innovation is the semiprone position. Because laparoscopic surgery can be performed with the abdominal wall closed, an advancement of laparoscopic surgery is that the patient position can undergo large conversion as needed. The major advantage of the semiprone position over the supine or semilateral position is that it ensures a better view and significantly improves the operability in the right lateral to posterior side of the liver, which allows for liver mobilization from the IVC and parenchymal dissection around the hepatic vein while minimizing bleeding. In the semiprone position, not only the right lobe of the liver but also the organs surrounding the hepatoduodenal ligament, such as the stomach, duodenum, and colon, slip downward to the left lower abdomen. The liver will never slip downward unless mobilized. This condition allows for a sweeping view of the hepatic hilar area from center to right dorsal, which must be visualized to perform hilar dissection for the exclusion of portal pedicles. In particular, looking up from the dorsal aspect, Rouviere’s sulcus is easily visible in the center of the field.

The second innovative technique is the intrahepatic extrafascial approach. The extrafascial approach was developed by Couinaud [5]. In the extrafascial approach alone, a whole pedicle is rarely dissected directly; instead, the left medial pedicle when the umbilical fissure is open, often the whole left pedicle and occasionally the right lateral pedicle when visible in Rouviere’s fissure are dissected. Takasaki et al. [6] developed the extrafascial approach for the right liver as a Glissonian pedicle transection method in 1986. In this method, because the full length of the primary branches and the origin of the secondary branches are located extrahepatically, the origin of the three segmental branches can easily be taped outside the liver without having to incise the liver parenchyma. In 1990, Galperin and Karagiulian [7] used small incisions on the inferior surface of the liver, tunneling into the liver parenchyma until the sheath was found. In fact, it is not easy to tape each secondary branch without any division of the liver parenchyma because the sense of touch is not utilized in laparoscopic surgery. In particular, because the branching of the right branch is not consistent, taping only the right branch is not easy. Rouviere’s sulcus is an important landmark identified by Henri Rouviere in 1924 and is used as a reference point to guide the commencement of safe dissection [8, 9]. It is a cleft in the liver running to the right of the hilum, anterior to the caudate process, which contains the right portal pedicle. It is a useful demarcation for the division between S5 and S6 of the liver. Rouviere’s sulcus has been found (open) in more than 90 % of patients in Japan [10]. Rouviere’s sulcus is located at the right end of the hilar plate and generally shifts to the posteroinferior aspect of Glisson’s sheath. Therefore, the posteroinferior aspect of Glisson’s sheath can be easily isolated at the open Rouviere’s sulcus. The liver parenchyma may be divided by following Glisson’s sheath extrafascially, which should reach any branches of the right liver. All portal pedicles lead to Rouviere’s sulcus, which corresponds to the umbilical fissure pointed out by Couinaud in the left liver [11]. This method leads to selective hepatic vascular exclusion, the ability to perform partial resection with minimal bleeding and ischemic injury, and the ability to perform anatomical resection, including major hepatectomy such as posterior sectionectomy and right hemihepatectomy.

In conclusion, PLH in the semiprone position for tumors present in the dorsal anterior sector and posterior sector reduced intraoperative bleeding and shortened the postoperative hospital stay. This method is safe and expands the indications for laparoscopic liver resection for tumors.

## Electronic supplementary material

The video demonstrates a pure laparoscopic posterior segmentectomy with the patient in the semiprone position.

Below is the link to the electronic supplementary material.

Video, Supplemental Digital Content 1 (MP4 73259 kb)

## References

[1] Ikeda T, Yonemura Y, Ueda N, Kabashima A, Shirabe K, Taketomi A (2011). Pure laparoscopic right hepatectomy in the semi-prone position using the intrahepatic Glissonian approach and a modified hanging maneuver to minimize intraoperative bleeding. Surg Today.

[2] Kazaryan AM, Rosok BI, Marangos IP, Rosseland AR, Edwin B (2011). Comparative evaluation of laparoscopic liver resection for posterosuperior and anterolateral segments. Surg Endosc.

[3] Cho JY, Han HS, Yoon YS, Shin SH (2008). Experiences of laparoscopic liver resection including lesions in the posterosuperior segments of the liver. Surg Endosc.

[4] Yoon YS, Han HS, Cho JY, Ahn KS (2010). Total laparoscopic liver resection for hepatocellular carcinoma located in all segments of the liver. Surg Endosc.

[5] Couinaud CM (1985). A simplified method for controlled left hepatectomy. Surgery.

[6] Takasaki K, Kobayashi S, Tanaka S, Saito A, Yamamoto M, Hanyu F (1990). Highly anatomically systematized hepatic resection with Glissonean sheath code transection at the hepatic hilus. Int Surg.

[7] Galperin EI, Karagiulian SR (1989). A new simplified method of selective exposure of hepatic pedicles for controlled hepatectomies. HPB Surg.

[8] Rouviere H (1924). Sur la configuration et la signification du sillon du processus caude. Bulletins et Memoires de la Societe Anatomique de Paris.

[9] Hugh TB, Kelly MD, Mekisic A (1997). Rouvière’s sulcus: a useful landmark in laparoscopic cholecystectomy. Br J Surg.

[10] Kawarada Y, Das BC, Taoka H (2000). Anatomy of the hepatic hilar area: the plate system. J Hepatobiliary Pancreat Surg.

[11] Couinaud C (1981). Controlled hepatectomies and exposure of the intrahepatic bile ducts. Anatomical and technical study.

